# Human reliability analysis in maintenance and repair operations of mining trucks: A Bayesian network approach

**DOI:** 10.1016/j.heliyon.2024.e34765

**Published:** 2024-07-18

**Authors:** Ali Reza Zaker Hossein, Ahmad Reza Sayadi, Mohammad Javad Rahimdel, Mohammad Reza Moradi

**Affiliations:** a Department of Mining Engineering, Faculty of Engineering, Tarbiat Modares University, Iran; b Department of Mining Engineering, Faculty of Engineering, University of Birjand, Birjand, Iran; c Goharzamin Mining and Industrial Company, Iran

**Keywords:** Maintenance, Mining trucks, Human reliability, Bayesian network, Fuzzy set theory

## Abstract

Failures in mining machinery can abruptly halt mineral production and operations, emphasizing the indispensable role of humans in maintenance and repair operations. Addressing human errors is crucial for ensuring a safe and reliable system, particularly during maintenance activities where accidents frequently occur. This paper focuses on evaluating Human Reliability (HR) to enhance activity implementation effectiveness. Given the challenge of limited and uncertain data on human errors, this study aims to estimate the probability of human errors using Bayesian networks (BN) under uncertain parameters. Applying this approach to assess HR in the maintenance and repair operations of mining trucks at Golgohar Iron Ore Mine in Iran, the study identifies critical factors influencing error occurrence in a fuzzy environment. The results highlight key factors impacting human error and offer insights into estimating HR with minimal human intervention.

## Highlights

• Factors influencing the behavior of maintenance technicians were identified.
• A Bayesian network approach estimates a human error probability of 11 %.
• Job-related and organizational factors significantly impact human error occurrence.
• Maintenance scheduling and repetitive tasks are critical nodes contributing to HEs.

## 1 Introduction

Nowadays, the increasing complexity and automation in various industries have heightened the importance of human involvement in ensuring the correct operation and reliability of systems [[1], [2], [3], [4]]. Any occurrence of human error can significantly impact the performance of a system. Human error is a critical hazard in the work environment and is recognized as a leading cause of occupational accidents [[5], [6], [7]].

Maintenance activities expose maintenance technicians and other workers to various physical hazards, such as noise, mechanical vibrations, excessive temperature, radiation, gases, and high physical workload, as well as psychosocial hazards like time pressure placed on maintenance crews to complete tasks quickly. Additionally, maintenance tasks require carrying heavy materials, bending, twisting, kneeling, reaching, pushing and pulling, and working in confined spaces, all of which can lead to serious injuries [[8], [9], [10], [11]]. Human errors are a critical factor in assessing human reliability and reducing the likelihood of workplace accidents. Therefore, it is crucial to identify, predict, and evaluate human errors, and provide suitable control solutions to decrease their incidence. Human behaviors in work environments are affected by environmental conditions, level of technical knowledge, management restrictions, and work environment. It is necessary to focus on evaluation, analysis, and mitigation of human errors in all operational systems, especially those systems in which the occurrence of human error can have disastrous consequences.

The mining industry is a high-risk work environment that exposes employees to a high level of workplace health and safety hazards [[12], [13], [14], [15]]. Human error is one of the main causes of mining accidents and poses a significant threat to the work environment. Classification of errors and evaluating their probability of occurrence plays a precious role in providing helpful insight into the causes of errors and possible ways to prevent them. Human error has become an essential issue in the mining environment because of an increasing number of accidents and costs [1,16,17]. The absence of adherence to safety norms, inappropriate usage of control tools, and lack of sufficient knowledge about equipment and processes have a significant impact on the happening of accidents in the mining industry. Utilizing proper techniques to identify, predict, and investigate potential human errors, as well as seeking suitable control solutions, offers the most feasible options to mitigate and reduce the happening of errors and to minimize system vulnerabilities.

In our time, some researchers have studied the influential factors behind human error in mining operations. Li et al. [18] categorized and identified human errors in shield machine operation during tunneling construction using the Technique for the Retrospective and Predictive Analysis of Cognitive Errors (TRACEr). The TRACEr method together with the hierarchical task analysis was applied for the decomposition of the shield machine operation tasks into the combinations of activities. Then, the Phoenix data mining technique is used to develop the realistic structure of human errors by creating an interface among activities and their cognitive functions. Kumar et al. [19] estimated the human error level of underground coal miners in India. In this study, the fuzzy logic theory was used for quantifying the human error level in different mining activities regarding past accident reports. To this, the accident report was translated to the fuzzy set of human error, and then the fuzzy sets of errors were developed to calculate the error rate. The reviewed study showed that handling and transportation of materials were the most insecure activities. Sun et al. [20] applied the Bayesian network method to assess the human reliability of intelligent hoist operation in a coal mine. The performance shaping factors (PSFs) were identified based on the cognitive behavior model of the hoist system operators. Subsequently, a Bayesian network was developed to depict the relationships between various PSFs. Moreover, different influencing factors of human reliability were obtained. The results of this study revealed that the interdependence of cognitive errors had adverse effects on the human factor reliability. This study found that several factors, such as complexity, workload, human-machine interface, education, and training, significantly influence human reliability. In a study by Mohammadfam et al. [21], the influence of human error on surface mine design was investigated using multi-criteria decision-making methods. The fuzzy Delphi method was used to identify the variables affecting design errors, and the DEMATEL method was applied to determine cause-and-effect relationships between the variables in a fuzzy environment. The results indicated that technical knowledge and experience were the most influential factors contributing to design errors.

In another study, Taheri et al. [22] evaluated the human error in mine workforces during the COVID-19 pandemic. In the mentioned study, the Cognitive Reliability and Error Analysis Method (CREAM) was applied to evaluate human errors in mining, crushing, processing, and supporting subunit operations of miners in a copper mine in Iran. In the obtained results, it was found that mine workers were at a higher risk of human error due to poor working conditions. Aliabadi et al. [23] conducted a study on human errors in the blasting operation of iron ore mining. In their study, they identified all possible tasks in the drilling and blasting operation, and then used the Best-Worst method to determine the weight of each task. The Human Error Assessment and reduction technique (HEART) was applied to estimate the probability of human error in the blasting process at an iron ore mine in Iran. The study identified the most dangerous task and recommended measures to improve human reliability. In a recent research conducted by Li [24], the risks of human errors in crane operations were prioritized using the Failure Mode and Effects Analysis (FMEA) approach within a fuzzy environment. The study integrated weights of occurrence, severity, and detection risk factors using the Enhanced Combination Weighting Model of Game Theory (ICWGT) and determined the ranking of human error risks using the Cumulative Prospect Theory (CPT).

Based on the aforementioned studies, it is evident that significant research has been conducted to identify and prevent the causes of mining accidents. Additionally, extensive efforts have been made to understand the factors contributing to human error in mining. Nevertheless, there is a lack of comprehensive research focusing on assessing human errors during the maintenance and repair of mining equipment, particularly within Iranian mining operations.

Mining operations consist of four main stages: drilling, blasting, loading, and transporting minerals. Transportation of minerals accounts for approximately 50–60 percent of total operating costs of mines. It is crucial to utilize reliable transportation methods with low operating costs over their entire operational lifespan [25,26]. In open-pit mining operations, various transportation systems such as trucks, conveyors, semi-mobile, and mobile crushers are deployed to transport broken ore from the mine working faces to the crushing plant. Trucks are considered an economical, flexible, and efficient mode of transportation. More efficient and safer transportation systems allow for the exploitation of mines with the lowest operating costs. This requires equipment with low downtime and minimal inspection, servicing, and replacement needs. A mining truck is a complex vehicle with various interconnected parts. Any failure in its subsystem, component, or part can cause the truck to fail, disrupting the mineral transportation process and reducing mine productivity. Therefore, it is vital to maintain the truck at a high level of availability.

The maintenance and repair operations of mineral transportation machines have a significant impact on the economic efficiency of the mine. When equipment fails due to insufficient maintenance, it results in high operating costs for the mine. These costs can include labor, consumables, spare parts, as well as the costs associated with machine downtime and loss of production. When maintenance activities are carried out by experienced and trained technicians to a high standard, the likelihood of machine failure due to human errors is significantly reduced. Poor maintenance and incomplete or unprincipled repairs can lead to a decrease in equipment reliability and availability. Therefore, identifying factors that affect the behavior of maintenance and repair technicians can reduce the likelihood of human errors and equipment failure.

It is crucial to reduce human errors in the maintenance and repair of mining trucks as these directly impact the costs incurred from damage to employees, equipment, especially trucks, and overall productivity. Failure to address this issue results in increased production costs and decreased profits for mining operations. Therefore, human performs a vital function in the maintenance and repair operation of mining trucks. This research intends to analyze the human reliability in the maintenance and repair operations of mining trucks and trying to identify the factors affecting the occurrence of human error in mining environments. For this purpose, the Bayesian networks model is used in the field of probabilistic reasoning. It is converted into a connected tree based on the reasoned probabilities to evaluate human reliability in the maintenance and repair process of trucks. One of the key advantages of Bayesian networks, which has increased their appeal compared to other methods, is their ability to model accurately even with small amounts of data. They can also calculate the likelihood of human error and estimate human reliability. In light of this, the main questions of the current study can be summarized as follows.

- What factors affect the efficiency of the maintenance operators of the mining heavy-duty trucks?
- What is the probability of human error in implementing each maintenance activity?
- What are the practical solutions to improve the maintainability of mining trucks with an emphasis on improving human reliability?
- What is the impact of the factors related to the maintenance and repair of mining trucks in the occurrence of individual errors during the repair and maintenance operations?

The findings of this research are valuable for mine managers as they can help in identifying the factors that contribute to human error in the maintenance and repairs department. By identifying influential factors for human error in the maintenance and repair process it is more reachable to reduce the probability of human error occurrence during maintenance and repairs procedures. This is also useful for estimating human reliability using a fuzzy-based Bayesian network approach.

## 2 Research methodology

Human factors have not been adequately considered in the physical design of equipment and the development of processes related to the maintenance of mining machinery. Therefore, it is necessary to study human actions in operational tasks to analyze human reliability as part of reliability. Generally, human reliability analysis (HRA) approaches are developed in three stages. The first generation of human reliability methods was introduced in 1970–1990, the focus of which was on human error likelihoods and human operational errors [27]. Technique for Human Error-Rate Prediction (THERP), Human Cognitive Reliability (HCR), and Human Error Assessment and Reduction Technique (HEART) are the first-generation approaches in which traditional methods such as the event tree analysis are used for the estimation of the human error probability [28]. The second generation of reliability approaches was presented between 1990 and 2005, with a focus on the factors that contribute to performance and cognitive processes [29]. Performance shaping factors can be internal or external and generally encompass everything that affects human performance, such as workload, stress, sociological concerns, psychological concerns, illness, etc. The second-generation methods describe the main sources of specific erroneous human actions or the contexts in which human errors occur. Cognitive Reliability and Error Analysis Method (CREAM), Standardized Plant Analysis Risk (SPAR), and A Technique for Human Event Analysis (ATHENA) are examples of well-known and widely used methods. In this set of techniques, the human error probability is quantified based on error-inducing conditions or context. Finally, the third generation of reliability analysis approaches, which began in 2005 and continues today, concentrates on performance-shaping factors, relationships, and dependencies [30]. In the third-generation methods, artificial intelligence and computer-aided simulation techniques are applied to predict human error probabilities that are still under development.

Nowadays, various HRA methods have been employed to evaluate common performance conditions and assess human reliability in different industries. Some previous studies did not consider the interaction between common performance conditions and assumed unrealistic independence between human factors and associated actions [20,[30], [31], [32]]. However, the capability of updating the probability is essential to reanalyze the posterior error probability instantly regarding new information. In such uncertain circumstances and by capturing conditional independence interactions among interrelating variables, probabilistic graphical models like BNs could be applied. Some literatures have employed fuzzy-based Bayesian Networks (BNs) CREAM without establishing the conditional probabilities of BNs through a straightforward methodology [[33], [34], [35]]. In this study, to address these limitations, the Analytical Hierarchy Process (AHP) is incorporated into BNs as an intelligent tool to analyze various factors and rank them based on their influence under a fuzzy environment. The hierarchical structure is utilized to decompose, organize, and control complexity of decision-making. Fuzzy set theory quantifies the conditional probabilities in an effective and straightforward manner to improve the accuracy of error estimation and achieve better risk discernment. Then, the BN probabilistic graphical model is applied to quantify human error probability. This paper aims to utilize the mentioned methodology to assess human reliability in the maintenance and repair department of the mining transportation system as a case study at Golgohar Iron Ore Mine in Iran.

**Fig. 1:**
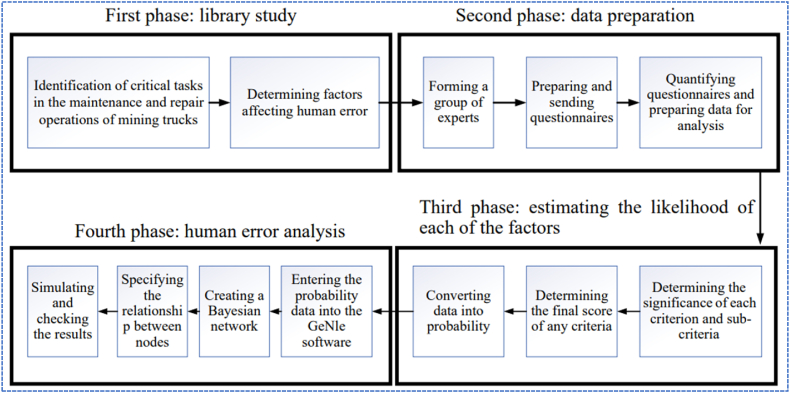
Scheme of research methodology.

Fig. 1 shows scheme of research methodology. In this way, influential factors on human error occurrence were identified and converted into a connected tree based on the reasoned probabilities. The field research method was utilized to gather the opinions of experts and review studies on human error. This approach involved analyzing and discussing the factors that influence human reliability and the consequences of human error during maintenance and repair operations of mining trucks. In the first phase, literature studies such as identifying critical tasks in the maintenance and repair operations of mining trucks and determining the factors affecting human error are performed. In the second phase, data is collected by forming a group of experts and preparing questionnaires. Data preparation is performed by quantifying the qualitative outputs from the experts' opinions. In the third phase, the importance level of each factor and sub-factor is determined, and the final score of each factor is then calculated. These final scores are then converted into probability values. In the fourth phase, a Bayesian network is constructed to analyze human error and evaluate human reliability by examining the relationship among factors and subfactors. The following section provides a detailed description of the research methodology.

### 2.1 Estimating the probability of each effective factor on human error

**Fig. 2:**
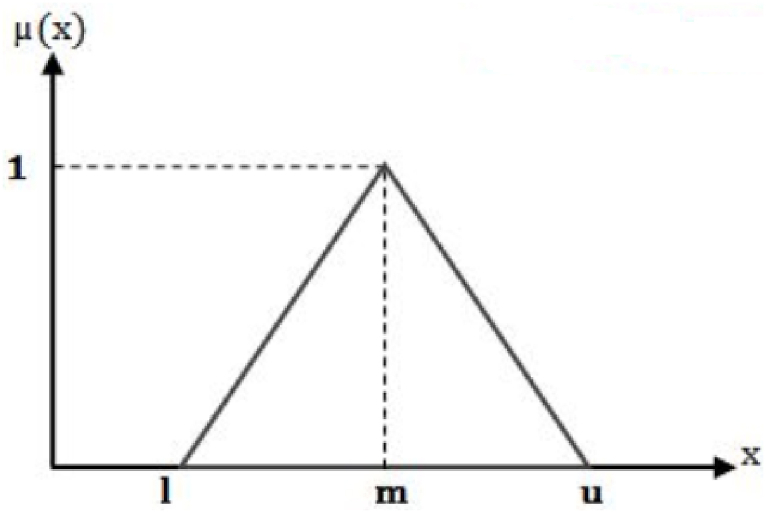
A triangular fuzzy number with a membership function.

The importance level of each decision-making factor is determined by the experts' experiences and knowledge or using the available quantitative data about the problem. In most cases, the information is insufficient and uncertain. In such situations, the experts’ judgments are very beneficial and provide a better view to the decision-makers. However, the experts' judgments are always associated with uncertainties. There are different methods to overcome these uncertainties. Fuzzy set theory is one of the effective solutions for this problem. In Fuzzy theory, to overcome the problems of classical methods, the importance degree of each factor is evaluated by fuzzy numbers instead of crisp values [36]. A fuzzy set is an extension of a crisp set, which allows for partial membership values varying between 0 and 1. There are different ways to display numbers in fuzzy sets. Fig. 2 shows an example membership function (μ(x)) of a triangular fuzzy number [37]. A prevalent type of fuzzy number in use is the triangular fuzzy number (TFN), represented as (*l*,*m*,*u*), where l ≤ m ≤ u. The parameters l, m, and u correspond to the lower limit demonstrating the minimal value, the middle value indicating the most optimistic or central value, and the upper limit signifying the maximal value achievable.

In order to gather and analyze the data points related to each factor for assessing human error, we need to create questionnaires. The number of questions in these surveys should match the number of influencing factors at each level of the hierarchical structure. The research model comprises two main levels: the primary factor (first level) and the sub-factor (second level). Following this, qualified experts review and complete the questionnaires. The people with sufficient knowledge and experience about the status of maintenance and repair of transportation systems were involved in the survey process. The selected experts were the officials, operators, repairmen, and technical engineers of the repair and maintenance department who are entirely related to the truck repair and maintenance operations. Table 1, Table 2 show the expressions used to gather qualitative opinions from experts. The inconsistency ratio of the matrices for the sub-factors related to each first-level factor was calculated using the Expert Choice software [38] to assess the reliability of the pairwise comparison questionnaire.

**Table 1:** Five-point scale to determine the weight of man factor.

Corresponding fuzzy set	Importance	Preferred value
(0,0,0.25)	Very low	1
(0,0.25,0.5)	Low	2
(0.25,0.5,0.75)	Medium	3
(0.5,0.75,1)	High	4
(0.75,1,1)	Very high	5

**Table 2:** Fuzzy set to express preferences between the sub-factor in the pairwise comparison matrix.

Description	Comparison status of *i* vs. *j*	Preferred value	Corresponding fuzzy set
Factors *i* and *j* have the same importance (no preference)	Equal importance	1	(1, 1, 1)
Factor *i* is slightly more important than *j*	Relatively more important	3	(2,3,4)
Factor *i* is more important than factor *j*	More important	5	(4,5,6)
Factor *i* is much more preferable than factor *j*	Much more important	7	(6,7,8)
Factor *i* is absolutely more important than *j* and is not comparable to *j*	Absolutely more important	9	(8,9,10)
Intermediate values	Somewhat high/strong/good	2,4,6,8	(1,2,3), (3,4,5), (5,6,7), (7,8,9)

After completing the questionnaires, the fuzzy sets shown in Table 1, Table 2 are utilized to quantify the qualitative opinions gathered from the experts. Let's assume that the number of experts involved in the survey process is denoted as *Z*, and the resulting fuzzy number from the opinion of expert *z* regarding the impact of each main factor on the first level of the hierarchy equals ${\tilde{s}}_{iz}=\left(a_{iz},b_{iz},c_{iz}\right)$. The consensus of the experts' judgments is calculated using the following equation:

(1) $${\tilde{S}}_{k}=\left(a_{k},b_{k},c_{k}\right)=\left(\min_{Z}\left\{a_{kz}\right\},\frac{\sum_{z=1}^{Z}b_{kz}}{Z},\max_{Z}\left\{c_{kz}\right\}\right),\left(z=1,2,\ldots ,Z\right)$$

Where, *a*_*k*_, *c*_*k*_, and *b*_*k*_ are the lower, upper, and average limits of the fuzzy number about factor *k* at the first level of the hierarchy. The fuzzy number obtained from the consensus of experts' judgment shows the fuzzy score of each factor affecting the likelihood of human error. The weighted average of scores is used to calculate the final score of each factor affecting human error by using the following equation:

(2) $$S_{j}=\sum_{i=1}^{n}\begin{array}{c} {s_{i}w}_{i},\sum_{i=1}^{n}w_{i}=1\; \end{array}$$

Where, *S*_*j*_ is the final score of the influencing factor *j* in the second level, *n* denotes the number of influencing factors connected to the influencing factor *i* in the second level, and *W*_*i*_ represents the weight of the influencing factor *i* in the second level.

The weight of each influencing factor in the second level indicates the extent of their influence on influencing factor *i* in the first level. There are various techniques to estimate the weights of influential factors. Here, the Analytical Hierarchy Process (AHP) is utilized in a fuzzy environment to determine the weight of each sub-factor (the second level of the hierarchy).

#### 2.1.1 Fuzzy analytical hierarchy process

The Analytical Hierarchy Process (AHP) is a widely known multi-criteria decision-making technique used to assess the significance of decision criteria under uncertain conditions. AHP is based on a pairwise comparison presented by Saaty [39]. However, the existence of an unbalanced scale in judgments and uncertainty in pairwise comparisons are the main drawbacks of this method. The AHP under the fuzzy environment (fuzzy AHP, FAHP) was proposed to overcome such problems [40,41]. In this approach, the matrix of pairwise comparisons among factors is constructed by using fuzzy numbers. So far, the FAHP has been effectively and successfully used to solve mining problems such as evaluating the safety of coal mines [37,42,43], mining method selection [40,44], choice of the suitable plant species for mine reclamation [45], site selection for the construction of a processing plant [46], and haulage system optimization [41]. Fuzzy AHP steps are summarized as follows [47,48].

##### 2.1.1.1 Creating the pairwise comparison matrix

The fuzzy pairwise comparison matrix, denoted by Ã, is formed after calculating the fuzzy numbers resulting from the consensus of experts' opinions. The presented pairwise comparison matrix is illustrated below:

(3) $$\tilde{A}=\left[\begin{array}{cccc} 1 & {\tilde{a}}_{12} & \cdots & {\tilde{a}}_{1n}\\ {\tilde{a}}_{21} & 1 & \cdots & {\tilde{a}}_{2n}\\ \vdots & \vdots & \ddots & \vdots \\ {\tilde{a}}_{n1} & {\tilde{a}}_{n2} & \cdots & 1 \end{array}\right]$$

This matrix contains fuzzy numbers, ${\tilde{a}}_{ij}=1/{\tilde{a}}_{ji}$. In this study, fuzzy numbers having a five-point scale are applied to create the pairwise comparison matrix. The fuzzy numbers related to the membership functions for the linguistic variables are provided in Table 1, Table 2

##### 2.1.1.2 Calculation of the fuzzy synthetic extent values

After creating the pairwise comparison matrices, the synthetic extent values (*S*_*i*_) value, a triangular fuzzy number, is derived using Eq. (4).

(4) $$S_{i}=\sum_{j=1}^{m}M_{gi}^{j}{\left[\sum_{i=1}^{n}\sum_{j=1}^{m}M_{gi}^{j}\right]}^{-1}$$

In this regard, the terms $\sum_{j=1}^{m}M_{gi}^{j}$, $\sum_{i=1}^{n}\sum_{j=1}^{m}M_{gi}^{j}$, and ${\left[\sum_{i=1}^{n}\sum_{j=1}^{m}M_{gi}^{j}\right]}^{-1}$ are obtained from Eq.s (5) to (7), respectively.

(5) $$\sum_{j=1}^{m}M_{gi}^{j}=\left(\sum_{j=1}^{m}l_{j},\sum_{j=1}^{m}m_{j},\sum_{j=1}^{m}u_{j}\right)$$

(6) $$\sum_{i=1}^{n}\sum_{j=1}^{m}M_{gi}^{j}=\left(\sum_{j=1}^{m}l_{j},\sum_{j=1}^{m}m_{j},\sum_{j=1}^{m}u_{j}\right)$$

(7) $${\left[\sum_{i=1}^{n}\sum_{j=1}^{m}M_{gi}^{j}\right]}^{-1}=\left(\frac{1}{\sum_{j=1}^{m}l_{j}},\frac{1}{\sum_{j=1}^{m}m_{j}},\frac{1}{\sum_{j=1}^{m}u_{j}}\right)$$

Where, *l*_*i*_, *m*_*i*_, and *u*_*i*_ represent the components of the fuzzy numbers as the first, second, and third components, respectively.

##### 2.1.1.3 Calculating the degree of possibility

**Fig. 3:**
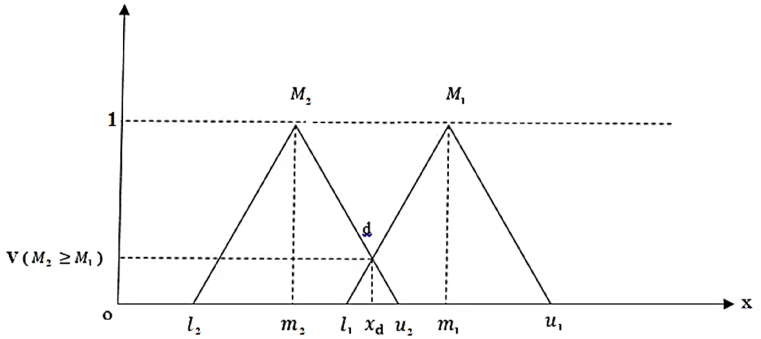
The magnitude of two triangular fuzzy numbers relative to each other.

After determining the values of *S*_*i*_, the relative degree of possibility of $S_{i}$ s is subsequently computed. If *M*_*1*_=(*l*_*1*_,*m*_*1*_,*u*_*1*_) and *M*_*2*_=(*l*_*2*_,*m*_*2*_,*u*_*2*_) depict two triangular fuzzy numbers, the possibility degree from *M*_1_ to *M*_2_ denoted as ($V\left(M_{2}>M_{1}\right)$) is calculated from Eq. (8). This value is shown in Fig. 3.

(8) $$V\left(M_{2}>M_{1}\right)=\left\{ \begin{array}{c} 1\;if\;m_{2}>m_{1}\\ 0\;if\;l_{1}<u_{2}\\ \frac{l_{1}-u_{2}}{\left(m_{2}-u_{2}\right)-\left(m_{1}-l_{1}\right)} \end{array}\right.$$

##### 2.1.1.4 Calculating the weight of each factor within the pairwise comparison matrices

The weight of each sub-factor in each pairwise comparison matrices is calculated from the following Eq.:

(9) *d’*(*A*_*i*_)*= Min V*(*S*_*i*_ *>* *S*_*K*_)*, k= 1, 2, …., n, k≠ i*

Therefore, the normalized weight vector is calculated as follows.

(10) *W’=* (*d’*(*A*_*1*_)*, d’*(*A*_*2*_)*, …., d’*(*A*_*n*_))^*T*^*A*_*i*_ (*i=1, 2, …, n*)

##### 2.1.1.5 Calculating the final weight of the sub-factor

The weight vector calculated in the previous step should be normalized to calculate the final weight of each sub-factor (Eq. (11)).

(11) *W=* (*d*(*A*_*1*_)*, d*(*A*_*2*_)*, …., d*(*A*_*n*_))^*T*^

#### 2.1.2 Converting fuzzy scores to the probability values

To determine the probability of each factor affecting human error, fuzzy scores are converted into fuzzy possibility scores (FPSs) representing the possibility of the root nodes as the following equation:

(12) $$FPS=\left(\frac{1-u}{1+\left(u-m\right)}\right)+1-\left(\frac{1-l}{1+\left(m-l\right)}\right)$$

Where, *l*, *m*, and *u* are the first, second, and third elements of a triangular fuzzy number.

Since using Bayesian networks is based on probability, it's essential to convert the possibility values to fuzzy probability values. For this purpose, the equations provided by Onisawa [49] are applied as follows [[50], [51], [52], [53]]:

(13) $$FFP=\left\{ \begin{array}{c} \frac{1}{10^{k}}\;FPS\neq 0\\ 0\;FPS=0 \end{array}\right.$$

(14) $$k={\left[\left(\frac{1}{FPS}\right)-1\right]}^{\frac{1}{3}}\times 2.301$$

Where, *FFP* is the fuzzy failure probability of each event obtained by expert evaluation and fuzzy theory, and *FPS* is fuzzy possibility scores.

### 2.2 Bayesian networks

A Bayesian network (BN) is a graphical model used to display probability distributions. There are generally three groups of probabilistic graph models: undirected probabilistic models, agent graphs, and directed models. Each of these models is used for different types of probability distributions [54]. In recent years, Bayesian networks, proposed by Thomas Bayes, are an efficient method to show the distribution of joint probabilities based on the conditional probability theory [55]. BNs are a set of nodes to display cause and effect relationships graphically. Hence, comprehending the cause-and-effect relationships between variables is straightforward. The attributes of each variable are depicted through one or more probability distributions. The BN is a powerful method that checks the risks and uncertainties in the models by providing probabilistic connections [56].

So far, extensive applications of Bayesian networks have been reported to solve different mining problems, including the prediction of the coal gas explosion [57], safety risk analysis [58,59], job-related damage analysis [23], prediction of rock-throwing [60], prediction of rock explosion [61], and analysis of machinery failure [26,62]. Bayesian networks are directed acyclic graphs, where the vertices represent random variables. In a Bayesian network, the set of vertices connected by a directed edge leading away from a specific vertex are known as the children of that vertex, and the set of vertices connected by a directed edge leading into the specific vertex are known as the parents of that vertex. A network in which no edge is called a null network and a network in which there is an edge between every vertex and all its vertices is called a complete network.

In a Bayesian network, the joint probability distribution of a set of variables is calculated by employing the following Eq.

(15) $$P\left(T\right)=\overset{n}{\underset{i=1}{∐}}(P\left(X_{i}|P_{a}\left(X_{i}\right)\right)$$

Where, $P_{a}\left(X_{i}\right)$ is the parent set of $X_{i}$ variables in the network that is defined by the following Eq.

(16) $$P\left(X_{i}\right)=\sum x_{i_{j\neq i}}P\left(T\right)$$

In BNs, a set of events is used to update previous events based on new observations. According to Bayes' theorem, the posterior or updated probability distribution for a variable is determined as per Eq. (17).

(17) $$P\left(X_{i}|E\right)=\frac{P\left(X_{i},E\right)}{P\left(E\right)}=\frac{P\left(E|X_{i}\right).P\left(X_{i}\right)}{\sum_{v}P\left(X_{i}|E\right).P\left(X_{i}\right)}$$

In the context where, $P\left(X_{i}|E\right)$ represents the posterior or updated probability given the evidence *E*, $P\left(X_{i}\right)$ denotes the prior probability of event $X_{i}$, $P\left(E|X_{i}\right)$ signifies the probability of events contingent on event $X_{i}$, P(E) stands for the predetermined posterior probability of evidence *E*, and $\sum_{v}P\left(X_{i}|E\right).P\left(X_{i}\right)$ symbolizes the joint probability distribution of evidence *E*.

In this approach it is possible to calculate the reliability using Eq. (18) with the probability of error [63].

(18) R=1−P(T)

Here, R represents the reliability factor, while *P*(T) denotes the probability of error.

### 2.3 Determining the importance degree of factors affecting human error

In a complex system, all components are not equally important. Accordingly, the factor affecting human error does not have the same effect on the probability of errors. Identifying the importance of factors is crucial in managing, controlling, and reducing human error. This research applied the Fussell–Vesely method to address the importance measure of each factor [64]. The importance measure of the factors in the Fussell–Vesely approach ($FVI\left(X_{i}\right)$) is calculated by using Eq. (19).

(19) $$FVI\left(X_{i}\right)=\frac{P\left(X_{i}\right)}{P\left(T\right)}$$

## 3 Case study

This section focuses on assessing human reliability during the maintenance and repair operations of mining trucks at Golgohar Iron Mine in Iran.

**Fig. 4:**
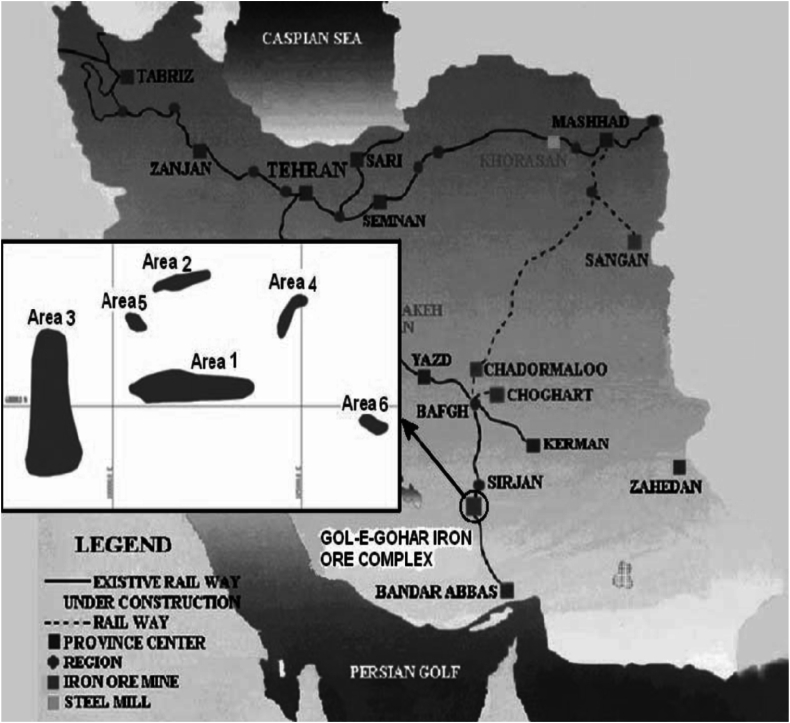
The location map of Golgohar Iron Ore Mine [66].

In order to achieve this objective, a comprehensive analysis of the factors influencing human error occurrence during the maintenance of mining trucks is conducted. The Analytical Hierarchy Process (AHP) within a fuzzy environment is employed to determine the degree of importance of each factor. Subsequently, a Bayesian network model is constructed to estimate human reliability in this context. The Golgohar Iron Ore Mine is situated 55 km southwest of Sirjan in the Kerman province of Iran, as depicted in Fig. 4. Within the Golgohar deposit, six distinct anomalies span an area of 10 km in length and 4 km in width, with a total estimated ore reserve of 1.135 billion tons. Notably, anomaly No. 3 is the largest among the iron ore anomalies at the Golgohar deposit, boasting a total estimated ore reserve of 616 million tons with an average grade of 54.3 % Fe [65]. To achieve this, all factors affecting human error occurrence in the maintenance of mining trucks are identified. The analytical hierarchy process under the fuzzy environment is used to obtain the importance degree of each factor. Then, a Bayesian network is developed to estimate the human reliability. Golegohar Iron Ore Mine is located 55 km southwest of Sirjan in the Kerman province of Iran (Fig. 4). The deposit comprises six separate anomalies within an area measuring 10 km in length and 4 km in width. Anomaly No. 3 is the largest among the iron ore anomalies at this deposit. The total ore reserve is estimated as 616 million tons having an average grade of 54.3 % [65].

Determining factors affecting human errors was based on library studies, field visits to the central mine maintenance and repair department, and collaboration and discussions with experts and employees of the mine maintenance and repair. In the first step of the research, to collect and determine the score of each factor in the evaluation of human error multiple-choice questionnaires were designed. The research identified two main levels: main factors (first level) and sub-factors (second level). Sixteen experts with knowledge and experience in truck repairs and failures in mine workshops were selected to participate in the trial. The educational backgrounds and experiences of the experts are summarized in Table 3.

**Table 3:** Working experience and educational level of experts.

Educational level	Frequency	Experience (year)	Frequency
Bachelor	9	4–8	5
Master	6	8–12	7
PhD	1	12–16	4

Based on the findings, the factors influencing human errors in the maintenance and repair operations of mining trucks can be grouped into five main levels: organizational, job, technical, environmental, and individual factors. These main factors, along with their corresponding sub-factors, are listed in Table 4.

**Table 4:** Factors affecting human reliability in maintenance and repair of the mining trucks.

Factor	Sub-factors	Sub-factors
Organizational (A)	A1	The description of assigned duties is not clear, complete, or accurate
	A2	Inadequate authority to carry out assigned missions and duties
	A3	Weakness of decision-making by managers or supervisors
	A4	Lack of accurate and up-to-date operational procedures and required work instructions
	A5	Improper safety and health equipment (fire extinguishers, signs, detergents)
	A6	Lack or ineffectiveness of on-the-job training
	A7	Lack of staffing in the repair shop
	A8	Lack of proper encouragement and punishment
	A9	Setting inappropriate times for maintenance and repair
	A10	Lack of necessary job security
Job (B)	B1	Variety of assigned tasks and activities
	B2	Simultaneous scheduling of the preventive and corrective maintenance activities
	B3	psychological aggression and emotional violence
	B4	Insufficient salary
	B5	The urgency of tasks and time constraints
	B6	Huge amount of work and assigned tasks
	B7	Lack of time to rest
	B8	Prolongation of repair operations after job hours
	B9	Pressures caused by work shifts, especially night shifts
	B10	Multiple, repetitive, and unnecessary maintenance operation
Technical (C)	C1	Weak design of tools and equipment
	C2	Lack or absence of required tools and equipment
	C3	Low-quality fuel and engine oil made in the country
Environmental(D)	D1	Noise
	D2	Humidity
	D3	Shock and vibration
	D4	Light
	D5	Dust
	D6	Air temperature
	D7	Toxic or smelly gases
	D8	Slippery ground
	D9	Space limitation of workspace to arrange tools and spare parts
Individual (E)	E1	Inadequacy of skills and experience with assigned tasks and activities
	E2	Inadequacy of education and expertise with the assigned job and duties
	E3	Inadequacy of physical condition with assigned activities and tasks (height, ability, age, etc.)
	E4	Haste to perform assigned duties
	E5	Feeling physically and mentally tired from doing daily tasks
	E6	Having stress in the workplace
	E7	Lack of motivation to perform tasks
	E8	Misconception about unnecessary items

To determine the weight of each factor, the fuzzy AHP technique was utilized. The normalized weight values of each sub-factor are given in Table 5. The consistency ratio of all sub-factors was calculated and given in Table 6. As per Saaty [39], an evaluation is considered reasonable when the consistency ratio falls below 0.1 in determining the importance degrees. In Table 7, all pairwise comparison matrices exhibit consistency ratios lower than 0.1 and therefore, there is consistency among the sub-factor of pairwise comparison.

**Table 5:** The normalized weight values of each sub-factor.

Code	Weight	Code	Weight	Code	Weight	Code	Weight
A1	0.102	B1	0.099	C1	0.308	D8	0.110
A2	0.102	B2	0.096	C2	0.358	D9	0.110
A3	0.101	B3	0.098	C3	0.332	E1	0.122
A4	0.098	B4	0.104	D1	0.112	E2	0.127
A5	0.096	B5	0.102	D2	0.110	E3	0.122
A6	0.097	B6	0.102	D3	0.108	E4	0.131
A7	0.102	B7	0.100	D4	0.114	E5	0.129
A8	0.100	B8	0.097	D5	0.111	E6	0.126
A9	0.098	B9	0.100	D6	0.111	E7	0.120
A10	0.099	B10	0.096	D7	0.110	E8	0.119

**Table 6:** Inconsistency ratio of pairwise comparison matrix.

Factor	Inconsistency ratio of the pairwise comparison matrix related to the factor
Organizational	0.020
Job-related	0.022
Technical	0.007
Environmental	0.022
Individual	0.021

**Table 7:** Failure probability of sub-factors.

Code	Probability	Code	Probability	Code	Probability	Code	Probability
A1	0.00302	B1	0.00317	C1	0.00158	D8	0.00299
A2	0.00302	B2	0.00323	C2	0.00135	D9	0.00299
A3	0.00303	B3	0.00319	C3	0.00146	E1	0.00287
A4	0.00306	B4	0.00311	D1	0.00296	E2	0.00280
A5	0.00309	B5	0.00315	D2	0.00299	E3	0.00286
A6	0.00308	B6	0.00314	D3	0.00301	E4	0.00276
A7	0.00302	B7	0.00317	D4	0.00293	E5	0.00279
A8	0.00304	B8	0.00320	D5	0.00297	E6	0.00282
A9	0.00308	B9	0.00316	D6	0.00298	E7	0.00289
A10	0.00306	B10	0.00322	D7	0.00298	E8	0.00290

Since the BN application is based on probability, it requires calculating the probability of each sub-factor at the first step. Results are given in Table 7. These results are used as Bayesian network inputs.

**Fig. 5:**
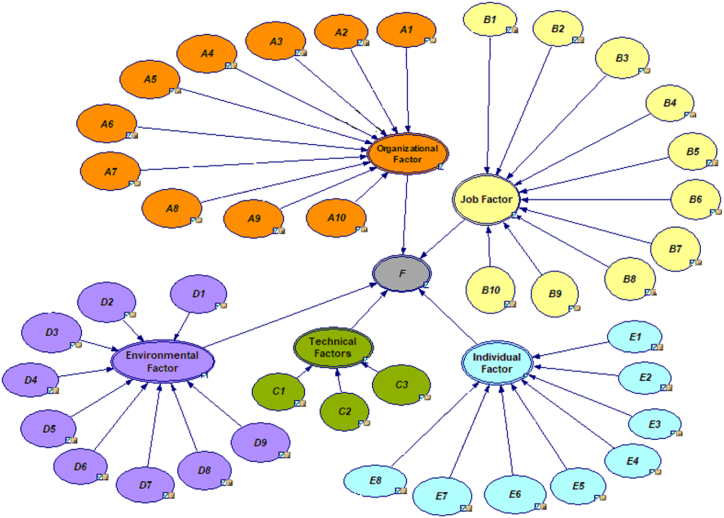
Bayesian network of factors affecting the occurrence of human error.

In the remainder of this section, a Bayesian network has been established to determine the human error probability. In this research, GeNIe software [67] was utilized to model and evaluate human reliability. To recognize all possible causes of errors and to specify critical sub-factors regarding the human error probability, a BN was developed and presented in Fig. 5. In this network, 40 root nodes (sub-factors in the second level), 5 intermediate nodes (main factors in the first level), and one top node (total error probability) are displayed. It is noted that with five main factors, there are 2^5^ = 32 possible states for all main factors. Similarly, there are 2824 possible states for all sub-factors. The numerous states result in extensive calculations for the conditional probability and load to the software. When plotting the BN and determining the relationships between the nodes, the probability data of each sub-factor is entered into the software from Table 6, and the error probability for each of the first-level factors and the total probability is calculated. The results are provided in Table 8. The human reliability in the maintenance and repair activities of the trucks is calculated and given in Table 8, as well. Regarding the results, the probability of human error is 11 %. This means that human reliability in the repair and maintenance of mining trucks is 89 %. A high level of human error indicates a situation with potential health and safety risks. Therefore, identifying key factors and proposing control measures are crucial steps in mitigating human error and establishing a safe work environment.

**Table 8:** Probability of error for first-level factors and total error.

Factor	Probability of human error in each factor	Total error
Organizational	0.030	0.11
Occupational	0.031	
Technical	0.050	
Environmental	0.026	
Individual	0.022	

**Fig. 6:**
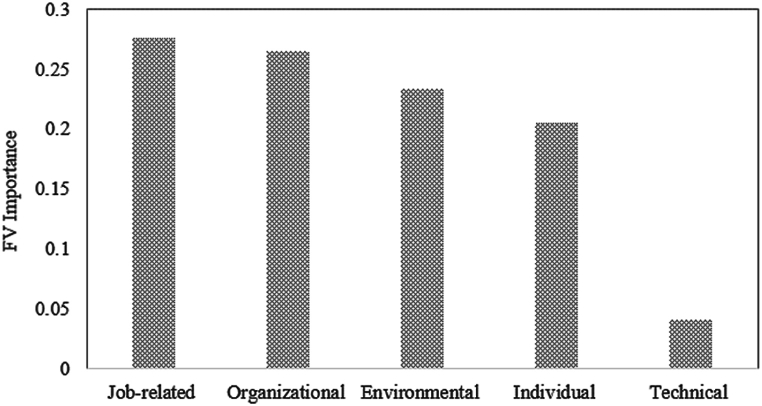
Importance of the main factors.

In the remainder of this section, the factors affecting human error will be identified. Additionally, practical solutions to improve people's health and safety will be proposed. As mentioned earlier, not all the factors influencing the occurrence of human errors have equal importance. Therefore, the identification of those factors that are critical from a human reliability perspective is crucial. This research applied the Fussell-Vesely method, as mentioned in section 2.3, to measure the importance of each factor affecting human errors. Results for the critical analysis of the first-level (factors) and the second-level (sub-factors) are revealed in Fig. 6 and Table 9, respectively.

**Table 9:** Importance of the sub-factors.

Code	Failure Probability	Rank	Code	Failure Probability	Rank
A1	0.0275	18	B1	0.0289	5
A2	0.0275	20	B2	0.0294	1
A3	0.0276	17	B3	0.0291	4
A4	0.0279	14	B4	0.0283	10
A5	0.0282	11	B5	0.0287	8
A6	0.0280	12	B6	0.0286	9
A7	0.0275	19	B7	0.0289	6
A8	0.0277	16	B8	0.0292	3
A9	0.0278	13	B9	0.0288	7
A10	0.0278	15	B10	0.0293	2
C1	0.0144	38	D8	0.0272	24
C2	0.0123	40	D9	0.0272	22
C3	0.0133	39	E1	0.0261	32
D1	0.0269	28	E2	0.0255	35
D2	0.0272	23	E3	0.0261	33
D3	0.0275	21	E4	0.0251	37
D4	0.0267	29	E5	0.0254	36
D5	0.0270	27	E6	0.0257	34
D6	0.0271	26	E7	0.0264	31
D7	0.0272	25	E8	0.0264	30

Regarding the results, job-related and technical factors have the highest and lowest impact on human error occurrence. The organizational, environmental, and individual factors have the second to fourth impact rating, respectively. As can be seen from the results and according to Table 9, among all job-related subfactors, the simultaneous scheduling of the preventive and corrective maintenance activities has a greater impact on human occurrence. The most appropriate action in this regard is managing the maintenance and repair time operations.

The multiple and repetitive maintenance tasks are placed in the second order, and the most appropriate measure to simplify and reduce this procedure is to manage the work process and simplify the method of performing the work tasks.

Improper safety and health equipment (fire extinguishers, signs, and detergents) were identified as the most probable cause of human errors among organizational sub-factors that require more attention to increase the health and safety level of the maintenance activities. Moreover, it is recommended to thoroughly document all repair procedures and enhance the expertise of technicians. Environmental sub-factors such as shock and vibration, as well as space limitations for organizing tools and spare parts, are of utmost importance. To reduce the adverse effects of the mentioned factors, the environment of the repair and maintenance shop should be improved. These measures might be an appropriate arrangement of tools and adequate ventilation and lighting based on standard instructions.

Misconceptions about unnecessary items and lack of motivation to perform tasks were the most important individual factors. Effective measures in this regard include adopting encouraging measures to increase the motivation of the maintenance workers and assigning tasks according to their expertise and experience.

Results of this study are beneficial for maintenance managers and designers to better identify the main factors influencing human error occurrence during mining truck repair and maintenance operations. This knowledge can help reduce the likelihood of errors and improve the operability of maintenance processes.

However, a limitation of the current study was the generation of expert opinions through an iterative process with controlled feedback. A formalized expert elicitation method, such as the Delphi approach, can be applied in future studies to elicit judgments on uncertainties, potentially leading to more robust results. Moreover, validating the quality and accuracy of the results obtained from this study was another limitation. To address this, the estimation of human errors can be validated by using simulator programs to generate more human error data for analysis. Data collection programs like SACADA (Scenario Authoring, Characterization, And Debriefing Application) and HuREX (Human Reliability Data Extraction) can be utilized in this approach.

## 4 Conclusions

This study investigated the factors influencing human errors in the maintenance and repair operations of mining trucks at Golgohar Iron Ore Mine, Iran, utilizing a Bayesian network approach. The analysis revealed that job-related and organizational factors significantly impact the likelihood of human error occurrence, with environmental, individual, and technical factors following suit. The calculated probability of human error stands at 11 %. Critical root nodes contributing to human errors include simultaneous scheduling of preventive and corrective maintenance, repetitive tasks, and improper use of control tools. To mitigate the risk of human errors during maintenance activities, recommended measures include effective maintenance planning, enhancing the quality and quantity of auxiliary equipment, improving workplace conditions, organizing tools properly, and providing clear instructions for maintenance tasks.

It is recommended that future research efforts should focus on evaluating human reliability in maintenance operations involving various mining equipment and assessing the effectiveness of proposed interventions in reducing the likelihood of human errors.

## Funding statement

There is no founding for this paper.

## Data availability statement

Data available on request from the authors.

## Additional information

No additional information is available for this paper.

## CRediT authorship contribution statement

**Ali Reza Zaker Hossein:** Writing – original draft, Resources, Methodology, Investigation, Formal analysis, Data curation. **Ahmad Reza Sayadi:** Writing – review & editing, Supervision, Methodology, Data curation, Conceptualization. **Mohammad Javad Rahimdel:** Writing – review & editing, Validation, Supervision, Resources, Methodology, Formal analysis, Data curation, Conceptualization. **Mohammad Reza Moradi:** Resources, Project administration, Data curation.

## Declaration of competing interest

The authors whose names are listed immediately below certify that they have no affiliations with or involvement in any organization or entity with any financial interest (such as honoraria; educational grants; participation in speakers’ bureaus; membership, employment, consultancies, stock ownership, or other equity interest; and expert testimony or patent-licensing arrangements), or non-financial interest (such as personal or professional relationships, affiliations, knowledge, or beliefs) in the subject matter or materials discussed in this manuscript.
